# Synthesis of Mesoporous ZnO Nanosheets via Facile Solvothermal Method as the Anode Materials for Lithium-ion Batteries

**DOI:** 10.1186/s11671-016-1244-9

**Published:** 2016-01-27

**Authors:** Xin Wang, Lanyan Huang, Yan Zhao, Yongguang Zhang, Guofu Zhou

**Affiliations:** Institute of Electronic Paper Displays, South China Academy of Advanced Optoelectronics, South China Normal University, Guangzhou, Guangdong Province 510006 China; Research Institute for Energy Equipment Materials, Hebei University of Technology, Tianjin, 300130 China; Tianjin Key Laboratory of Laminating Fabrication and Interface Control Technology for Advanced Materials, Hebei University of Technology, Tianjin, 300130 China; Institute for Advanced Materials (IAM), South China Academy of Advanced Optoelectronics, South China Normal University, Guangzhou, Guangdong Province 510006 China

**Keywords:** Lithium-ion battery, ZnO anode, Mesoporous ZnO nanosheet, Solvothermal, 82.47.Aa, 62.23.Kn

## Abstract

Mesoporous ZnO nanosheets are synthesized through a room temperature solvothermal method. Transmission and scanning electronic microscopy observations indicate that as-prepared ZnO hierarchical aggregates are composed and assembled by nanosheets with a length of 1–2 μm and a thickness of 10–20 nm, and interlaced ZnO nanosheets irregularly stack together, forming a three-dimensional network. Furthermore, large mesopores are embedded in the walls of ZnO nanosheets, confirmed by Brunauer-Emmett-Teller (BET) measurement. Accordingly, the resulting ZnO anode exhibits a high and stable specific discharge capacity of 421 mAh g^−1^ after 100 cycles at 200 mA g^−1^ and a good rate capability. Such electrochemical performance could be attributed to the multiple synergistic effects of its mesoporous nanosheet structure, which can not only provide a large specific surface area for lithium storage, but also favor the ion transport and electrolyte diffusion.

## Background

Lithium-ion batteries (LIBs) have received much attention in view of its applicability as popular power supplies in portable electronic devices [1]. A new demand of ever-growing electric vehicles (EVs) requires much higher specific capacities for both anode and cathode materials [2]. As a commercial anode material, graphite exhibits high coulombic efficiency and superior cyclability. However, owing to its relatively small specific capacity of 372 mAh g^−1^, intensive efforts are put on development of high-capacity anode materials with high efficiencies and long-term stability [3].

Among the various candidate materials, metal oxides have been considered as potential alternatives to replace commercial graphite as the anodes for LIBs due to their higher theoretical capacities [4]. Among various metal oxides, ZnO has received a particular interest as one of the most promising anodes due to its several obvious advantages, such as its high theoretical capacities of 978 mAh g^−1^, low-cost, facile preparation, and high chemical stability [5]. However, the practical use of ZnO as the anode for LIBs has been hindered by its large volume change during charge/discharge processes, which leads to high internal stress, electrode pulverization, and subsequent loss of electrical contact between the active material and current collector [6, 7].

Much effort has been dedicated to optimize and enhance the electrochemical performance of the ZnO anode in LIBs [5, 6]. Among them, nanostructured ZnO materials have been extensively explored with attempts to not only better accommodate large strains but also provide short diffusion paths for Li^+^ insertion/deinsertion [6]. Furthermore, mesoporous ZnO materials have been reported to exhibit good electrochemical performance for their high surface area, good accessibility of the pores, and large amount of electroactive sites [7].

Our belief at this point is that the combination of a nanostructure and mesoporous structure could present synergistic or cooperative effects to successfully overcome the effect of volume change and improve the electrochemical performance with excellent cycling stability and rate capability. Herein, we reported the preparation of mesoporous ZnO nanosheets via the solvothermal method. The physical and electrochemical properties of as-prepared ZnO as the anode for LIBs have been investigated.

## Methods

Mesoporous ZnO nanosheets were synthesized through a room temperature solvothermal method. The preparation techniques developed in this work allowed obtaining the ZnO via a low-cost and environment-friendly process, which takes place in aqueous media without the introduction of surfactants [8]. The typical preparation procedure is as described in the literature with slight modifications [8]: Firstly, 1.1 g Zn(AC)_2_ was dissolved into 80 mL DI water, and then 40 mL ethanol was added into the resultant Zn(AC)_2_ aqueous solution and stirred for 1 h. Then, 1 M of 80 mL NaOH aqueous solution was added with constant agitation at ambient temperature. After keeping stationary for 2 days, the precipitate was separated via filtration thoroughly with water and ethanol and dried at 60 °C for 12 h. Finally, the final product was obtained after aging at 300 °C for 30 h in air.

The crystalline phases of the as-prepared ZnO were characterized by X-ray diffraction (XRD, smart Lab, Rigaku Corporation, Cu Kα radiation). Nitrogen adsorption-desorption measurements were performed at 77 k by an Autosorb-iQ instrument (Quantachrome Instruments, USA). The specific surface area and the pore size distribution of the sample were evaluated via the Brunauer-Emmett-Teller (BET) method and Barret-Joyner-Halenda (BJH) method, respectively. Field emission scanning electron microscopy (SEM, S-4800, Hitachi Limited) and transmission electron microscopy (TEM, JEM-2100 F, JEOL) were applied to observe the surface morphology and the interior structure of the obtained products.

Coin-type cells (CR2025) with lithium metal as the counter and reference electrode were applied to investigate the electrochemical performance of samples at room temperature. The obtained ZnO samples were prepared as the working electrode. The ZnO powers were mixed with polyvinylidene fluoride (PVDF) as a binder and acetylene black conducting media of the weight ratio of 80:10:10 in 1-methyl-2-pyrrolidinone (NMP) solvent to prepare the electrode slurry. The slurry was uniformly coated onto nickel foam substrate with a doctor blade, and dried at 50 °C for 12 h. Circular disk electrodes 1 cm in diameter were prepared by punching under vacuum atmosphere. The active material loading in each electrode was about 2 mg cm^−2^. The separator was a polypropylene microporous film, and the electrolyte was 1 M LiPF_6_ dissolved in a mixed solution of dimethyl carbonate, diethyl carbonate, and ethylene carbonate (1:1:1 by volume). The cells were assembled in a glove box (MBraun) filled with argon atmosphere. Galvanostatic discharge/charge cycling tests were performed between 0.005 and 3 V at different current densities using a multichannel battery tester (BTS-5 V 5 mA, Neware). Between 0 and 3 V, cyclic voltammetry (CV) was carried out with a potentiostat (VMP3, Biologic) at a scanning rate of 0.5 mV s^−1^. All electrochemical measurements were performed at room temperature.

## Results and Discussion

**Fig. 1:**
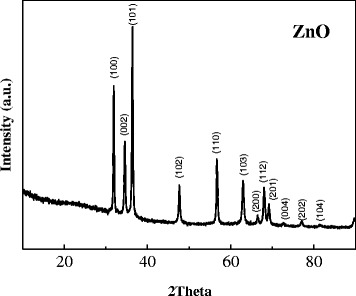
XRD diffractogram of the prepared mesoporous ZnO nanosheet

Firstly, Fig. 1 shows typical XRD patterns of the ZnO samples. All peaks in the patterns can be well indexed to hexagonal wurtzite ZnO (JCPDS card, No. 36-1451) [9]. No other detectable peaks from impurities are observed, indicating the high purity of as-prepared ZnO architectures. In addition, the strong and sharp reflection peaks suggest that the final product is highly crystalline.

**Fig. 2:**
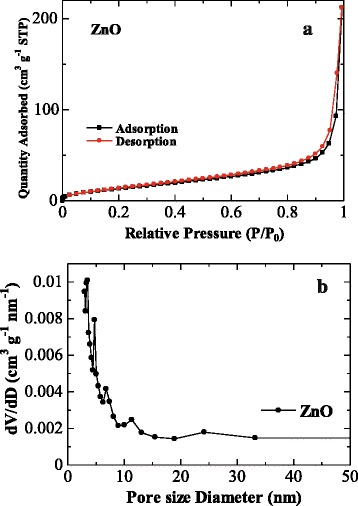
**a** Nitrogen adsorption/desorption isotherms of the mesoporous ZnO nanosheet. **b** Pore size distribution by using the BJH method of the mesoporous ZnO nanosheet

The specific surface areas and the porosities of the mesoporous ZnO nanosheet are characterized by measuring nitrogen adsorption isotherms as shown in Fig. 2. The BET surface area of the ZnO samples is calculated to be 56.7 m^2^ g^−1^, which is larger than that of commercial ZnO powders (~4–5 m^2^ g^−1^) [10]. Applying the BJH method, the calculated pore size distribution indicates that the sample contains mesopores with diameters in the range of 2–50 nm. This highly mesoporous structure could be very beneficial for assuming optimized morphology and fast kinetics for the ZnO anode, confirming the electrochemical results below.

**Fig. 3:**
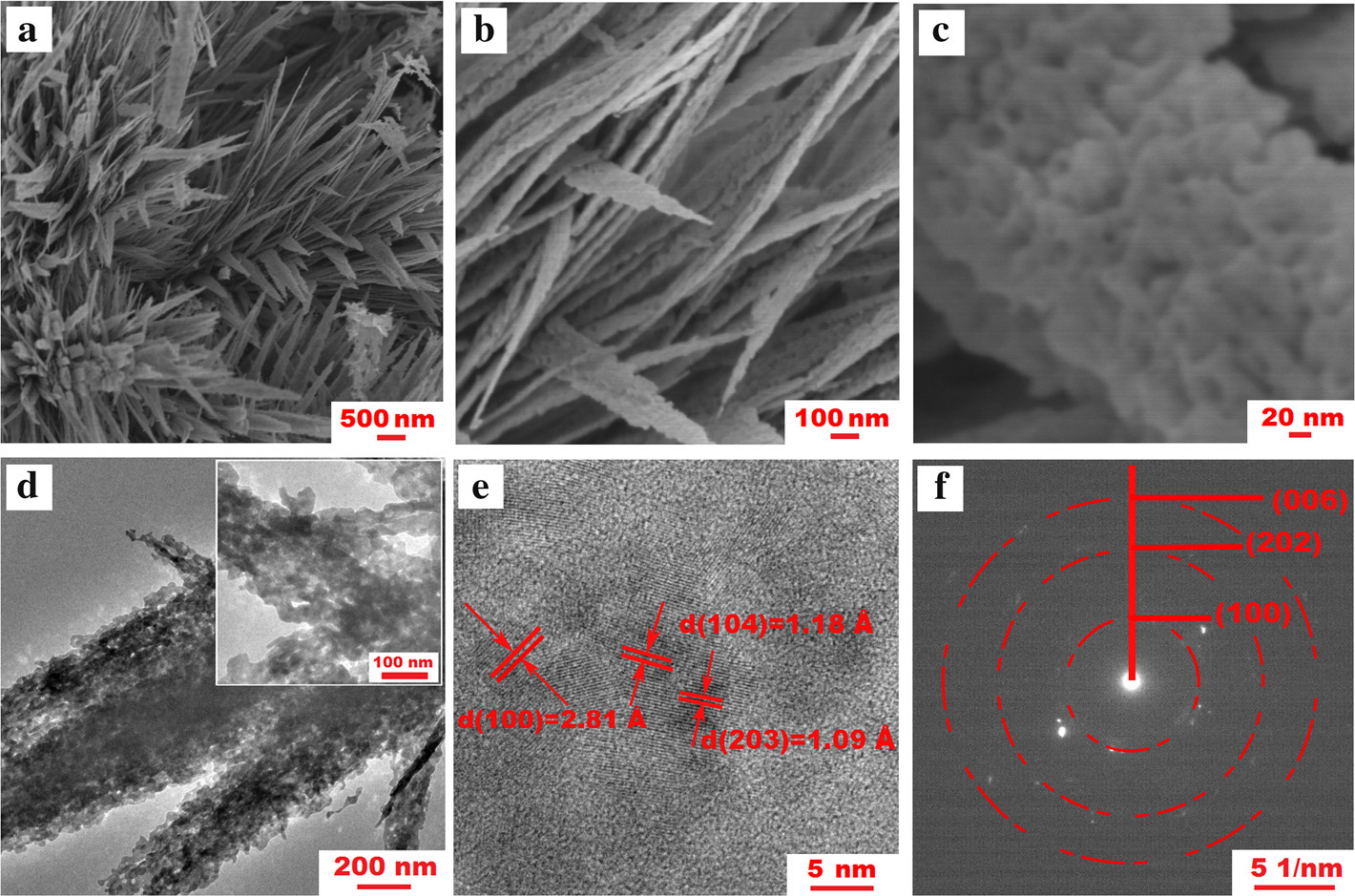
Structural characterization of the mesoporous ZnO nanosheets. **a**–**c** SEM micrographs of the ZnO architectures. **d, e** HRTEM images of the ZnO nanosheet. **f** SAED pattern of the ZnO nanosheet

The surface morphology of the ZnO architectures is depicted in the SEM micrographs (Fig. 3a–c). One can see that the ZnO hierarchical aggregates were assembled by nanosheets with length of 1–2 μm and thickness of 10–20 nm, and interlaced ZnO nanosheets irregularly stacked together, forming a three-dimensional network. At a high magnification (Fig. 3c), plenty of split-shaped pores are formed in the body of the microspheres. This resulting porous and loose architecture greatly favors the absorption of the electrolyte into the composite bulk, and its highly developed surface accelerates the occurrence of the electrochemical reactions of the ZnO/Li redox system [7]. Figure 3d shows high-resolution transmission electron microscopy (HRTEM) images of the as-prepared ZnO samples. One can see that a large amount of nanosized voids are present in the sheet, and so many pores in the nanosheets highly increase the surface area of the materials, positively affecting the overall electrochemical performance of the ZnO anode materials. In addition, the lattices of ZnO can be identified by the HRTEM image at a higher magnification (Fig. 3e), the well-resolved periodic lattice fringes with d-spacings of 2.81, 1.18, and 1.09 Å come from the (100), (104), and (203) planes of the ZnO (JCPDS card, No. 36-1451) [11]. The selected area electron diffraction (SAED) pattern of the ZnO, as shown in the Fig. 3f, consists of a few rings, which could be indexed with the hexagonal ZnO phase. This agrees well with the sharp peaks in the XRD patterns.

**Fig. 4:**
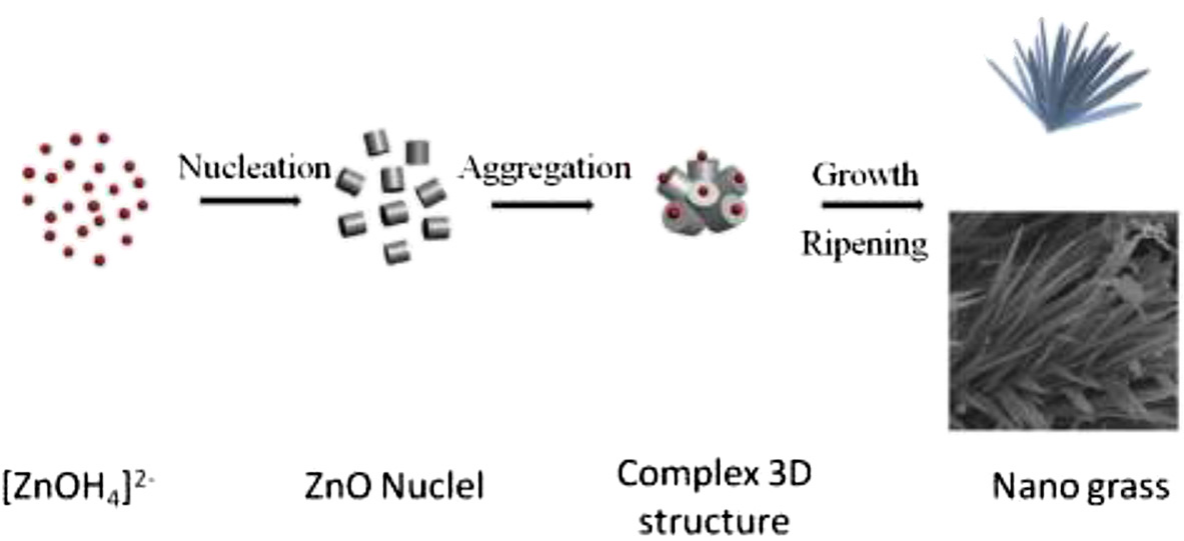
Schematic diagram of the possible growth mechanism for the formation of the ZnO architectures

Figure 4 depicts the possible formation process of the ZnO architectures. With the addition of NaOH solution, free Zn^2+^ is converted to the growth units [Zn(OH)_4_]^2−^. In the following reaction, small-sized ZnO nuclei are formed and tend to agglomerate the big particles. The aggregated nuclei surface offers active growth sites for subsequent growth, and a complex three-dimensional structure is kept in the following reaction [8]. With a high concentration of [OH^−^], as we adopted in this work, relatively fewer acetate anions exists in the solution to compete with the growth units [Zn(OH)_4_]^2−^ to occupy the growth sites, resulting in a preferential growth along the [0 0 0 1] direction to form a one-dimensional nanoneedle structure [8].

**Fig. 5:**
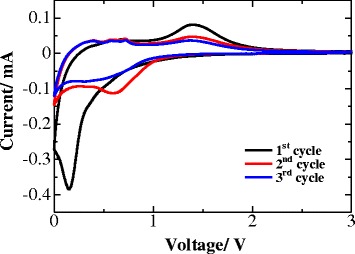
The initial three CV curves of ZnO anode at a scan rate of 0.5 mV s^−1^

The electrochemical property of the ZnO architectures as anode materials for lithium-ion batteries are examined by CV. Figure 5 shows the three initial cyclic voltammetry (CV) cycles of a typical cell containing a ZnO anode. One can see that at the initial cycle, there is a pronounced reduction process, which could be due to the reduction of ZnO into Zn, the formation of Li-Zn alloy, and the formation of the solid electrolyte interface (SEI) layer. In the subsequent cycles, several different oxidation peaks located at 0.3, 0.5, 0.7 and 1.4 V can be observed. The first three peaks indicate the occurrence of a multi-step dealloying process of Li-Zn alloy, which gives Zn metal (Zn^0^) through several stages, like LiZn → Li_2_Zn_3_ → LiZn_2_ → Li_2_Zn_5_, respectively [6, 12, 13]. And, the peak at 1.4 V is concerned with the decomposition of Li_2_O. Meanwhile, after the initial cycle, the current of the peaks remains unchanged upon the subsequent cycles, which confirms a good reversibility of the studied system.

**Fig. 6:**
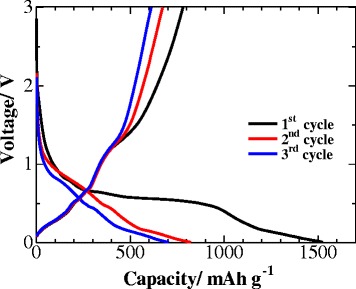
The galvanostatic discharge/charge profiles of the ZnO anode

The electrochemical performances of the ZnO architecture anode in galvanostatic charge/discharge tests with a current density of 200 mA g^−1^ are shown in Fig. 6. One obvious voltage plateau could be observed in the initial charge/discharge curves, which is in agreement with the CV results. The first discharge capacities (1523 mAh g^−1^) are much higher than the theoretical value of ZnO at 987 mAh g^−1^, which may derive from the SEI layer formation. After the initial cycle, no obvious capacity loss was observed, and the electrode could still maintain a reversible capacity of 701 mAh g^−1^ after three cycles. It is notable that, after the first cycle, the discharge and charge curves tend to overlap, suggesting a relatively steady state of the lithiation/delithiation process.

**Fig. 7:**
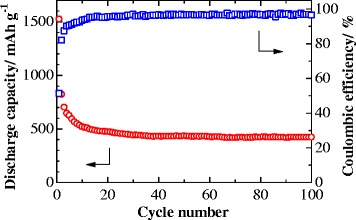
The cycling performance of the ZnO anode for 100 cycles

Figure 7 exhibits the discharge/charge cycling performance of the ZnO anode with a current density of 200 mA g^−1^ for 100 cycles. It can be seen that the discharge capacities drop quickly in the initial cycles and the cycling stabilizes after 10 cycles, and then is maintained at about 421 mAh g^−1^ up to 100 cycles, and a very small capacity fade of 17.7 % is observed over the last 90 cycles. Furthermore, the ZnO exhibits a high coulombic efficiency of about 95 % over 100 cycles. The good electrochemical performance could be attributed to the hierarchical porous structure of the ZnO architecture.

**Fig. 8:**
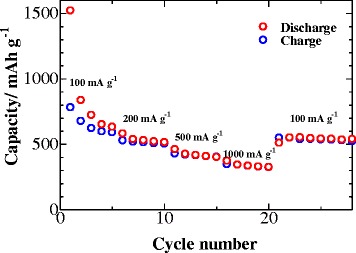
Rate performance of the ZnO anode

To further investigate the rate performance of the ZnO anode, a rate capability study was carried out at various rates as shown in Fig. 8. An increase in current rate from 100 to 1000 mA g^−1^ caused a drop in discharge capacity from about 632 to 325 mAh g^−1^. Notwithstanding, the discharge capacity is mostly recovered when the rate is switched back to 100 mA g^−1^. This is due to the formed three-dimensional hierarchical structure of the ZnO anode, providing negligible lithium-ion diffusion time and the favorable kinetics, which are important for enhancing the rate capability of the electrodes.

## Conclusions

In this work, we developed mesoporous ZnO nanosheets via a room temperature solvothermal method, which possesses both the merits of nanometer-sized building blocks and micrometer-sized assemblies. As-prepared ZnO hierarchical aggregates are composed and assembled by mesoporous nanosheets with a length of 1–2 μm and interlaced ZnO nanosheets irregularly stacked together, forming a three-dimensional network. When tested as an anode material for lithium-ion batteries, the initial specific discharge capacity was 1523 mAh g^−1^ at 200 mA g^−1^, with 421 mAh g^−1^ maintaining after 100 cycles, and 325 mAh g^−1^ at 1000 mA g^−1^. The good electrochemical performance could be attributed to the hierarchical porous sheet-like structure of the ZnO architecture, which can not only provide a large specific surface area for lithium storage, but also favor the ion transport and electrolyte diffusion.
